# Hesperidin associated with continuous and interval swimming improved biochemical and oxidative biomarkers in rats

**DOI:** 10.1186/1550-2783-10-27

**Published:** 2013-05-24

**Authors:** David Michel de Oliveira, Grace Kelly Zanotti Simoes Dourado, Thais Borges Cesar

**Affiliations:** 1 Department of Food and Nutrition, School of Pharmaceutical Sciences, UNESP, Rodovia Araraquara-Jau, km 1, Araraquara 14802-901, Brazil

**Keywords:** Hesperidin, Continuous swimming, Interval swimming, Blood serum biomarkers, Antioxidant, Rat

## Abstract

**Background:**

Citrus flavonoids, such as hesperidin, have shown therapeutic properties that improve hyperglycemia and insulin resistance, and decrease blood serum lipids and inflammation. The current investigation studied the effects of hesperidin supplementation associated with continuous and interval swimming on the biochemical parameters (glucose, cholesterol and triglycerides), and oxidative stress markers (TBARS and DPPH) in rats.

**Methods:**

The animals (n = 60) were randomly divided in six groups: negative (C) and positive control (CH) for hesperidin supplementation, and continuous or interval swimming without (CS and IS) or with hesperidin supplementation (CSH and ISH). Hesperidin was given by gavage for four weeks (100 mg/kg body mass) before the exercise. Continuous swimming was performed for 50 min with loads from 5% to 8 % of body weight from the first to fourth week, while interval swimming training was performed for 50 min in sessions of 1 min of swimming followed by 2 min of resting, carrying loads from 10% to 15, 20 and 25% from the first to fourth week. At the end of the experiment, blood serum samples were draw to perform analysis of glucose, total cholesterol, HDL-C and triglycerides. Oxidative biomarkers were evaluated by lipid peroxidation (TBARS) and antioxidant capacity assay (DPPH) of the blood serum.

**Results:**

There was a continuous decline of serum glucose from C (100%) > CH (97%) > CS (94%) > CSH (91%, p < .05), IS (87%, p < .05) > ISH (80%, p < .05), showing a combined beneficial effect of hesperidin and swimming. Also, continuous or intermittent swimming with hesperidin supplementation lowered total cholesterol (-16%, p < .05), LDL-C (-50%, p < 0.05) and triglycerides (-19%, p < 0.05), and increased HDL-C (48%, p < .05). Furthermore, hesperidin enhanced the antioxidant capacity on the continuous swimming group (183%, p < .05) and lowered the lipid peroxidation on the interval swimming group (-45%, p < .05).

**Conclusions:**

Hesperidin supplementation per se, or in combination with swimming exercise protocols, improved the biochemical profile and antioxidant biomarkers evidencing that the use of flavanones may enhance the health benefits promoted by exercise.

## Background

Regular practice of exercise has been recommended by health-care professionals as a coadjuvant element and a protective factor to control metabolic, hormonal, and cardiovascular parameters associated with the development of chronic diseases [1]. Variables such as type, quantity and intensity of the exercise can have crucial metabolic and physiologic effects. In this sense, continuous exercise is characterized by moderate to intense exercise of extended duration using fatty acids as the predominant energy source. On the other hand, interval exercise is defined as high intense exercise with passive or active pauses using glucose as the predominant source of energy [2]. Continuous and interval exercise protocols have been used as a strategy to control glucose and lipids of blood stream [3-7].

Exhaustive exercise and overtraining may increase the rate of free radical production to a level which exceeds the capacity of the cellular defense system, and consequently impairs the cell viability and initiates the damage on the skeletal muscle and promotes inflammation [8]. To minimize these negative effects, antioxidant supplements can be taken to attenuate the side-effects of exercise, and flavonoids in general can be used to improve the antioxidant capacity [9,10]. Previous studies in humans and animals, especially rodents, have demonstrated that hesperidin and its metabolites decrease blood serum glucose and lipids and neutralize markers of oxidative stress [11-14]. Although a body of evidence has shown these benefits, most of the mechanisms are still being explored [9,15-18].

The purpose of this study was to analyze the interaction of hesperidin and continuous or interval exercises, evaluated by potential changes on biochemical parameters, as glucose, cholesterol and triglycerides, and biomarkers of oxidative stress in rats, as lipid peroxidation (TBARS) and antioxidative capacity (DPPH). We compared the blood levels of glucose and lipids in rats submitted to continuous exercise and interval swimming protocols, and we also evaluated two oxidative biomarkers for both protocols plus the effect of hesperidin supplementation. The following hypotheses were tested: (1) The improvement of the blood serum variables by the continuous and interval swimming with hesperidin supplementation; and (2) the reduction of oxidative stress rate, promoted by continuous and interval exercises, by the antioxidant effects of hesperidin supplementation.

## Methods

### Reagents

Hesperidin supplement was obtained by Hyashibara, Japan, as glucosyl hesperidin, because of the higher bioavailability in comparison to the regular hesperidin compound. Biochemical analyses (glucose, triglycerides, cholesterol total, HDL-C) were determined using commercial kits (Labtest, Brazil) by Technicon RAXT chemistry analyzer (Bayer Diagnostic). LDL-C was determined according to Friedewald et al. [19]. Reagents for lipid hydroperoxide and antioxidant substances (TBARS and DPPH) were obtained from Sigma-Aldrich.

### Animals and experimental groups

Male rats *Wistar*, 12 weeks old, 300-450 g, were maintained in collective cages (5 rats/cage, 350 cm^2^/animal, 18 cm of height) on a normal light/dark cycle in a climate-controlled environment and fed with standard rodent chow diet and water *ad libitum*. The body weights were determined once a week. This study was approved by Ethics Committee on Animal Research at the University of Franca, Sao Paulo, Brazil (Protocol nº 0038/10).

Animals (*n* 60) were randomly divided into six groups (*n* 10), as follow: (1) Negative Control (C): no swimming and no supplement; (2) Positive Control (CH): no swimming plus hesperidin supplement; (3) Continuous Swimming (CS): continuous swimming and no supplement; (4) Continuous Swimming plus hesperidin (CSH): continuous swimming plus hesperidin supplement; (5) Interval Swimming (IS): interval swimming and no supplement; 6) Interval Swimming plus hesperidin (ISH): interval swimming plus hesperidin supplement.

### Hesperidin supplementation

Groups supplemented with the isolated flavonoid received glucosyl hesperidin diluted in saline (100 mg/kg body mass) by gavage for four uninterrupted weeks, thirty minutes before of the animals performed the exercise. The amount of glucosyl hesperidin was adjusted in accordance with the weight of each animal.

### Swimming protocols

The animals were trained on continuous or interval swimming during 50 min per day for four weeks, after one week of adaptation. Rats swam in square polypropylene tanks (5 rats/tank) filled with water (40 cm depth) at 27°C. They were randomly divided in 6 groups and 4 of the groups were subjected to swimming in either of two ways: continuous swimming or interval swimming. Continuous swimming was characterized by cyclical and uninterrupted movements between the arms and legs, using a predominance of the aerobic energy for 50 minutes, carrying a weight equal to 5% of their body in the first week, gradually progressing to 6, 7 and 8% on the second, third and fourth week [3]. Interval swimming training was performed for a 50 min total period, characterized by brief periods of high-intensity exercise (60 s) following by rest periods (120 s) on a submersed platform, using a predominance of anaerobic energy, carrying a weight equal to 10 % of their body in the first week, gradually progressing to 15, 20 and 25% on the second, third and fourth week. This protocol was adapted from Oliveira et al. [20].

### Biochemical analysis

One day after the experimental period the animals, fasted for 12 h, were decapitated by guillotine, the blood was collected and centrifuged to obtain serum, which was stored at -20°C. Serum glucose, total cholesterol, HDL-C and triglycerides were determined by commercial kits (Labtest, Brazil).

### Lipid hydroperoxide (TBARS assay)

Thiobarbituric acid-reactive substances (TBARS) assay was used to determinate the lipid peroxidation of the animals’ serum [21,22]. Two hundred mL of MDA standard (0; 1.25; 1.88; 2.50; 3.13; 3.75; 6.25 e 12.50 M) and serum sample were mixed with 200 μL of SDS and then 500 μL of staining reagent (5.3 mg/mL of TBA diluted in acetic acid 20%, pH 3.5) were vortexed and incubated at 100ºC for 60 min, and cooled on ice for 10 min. The standards and samples were centrifuged at 10,000 rpm for 10 min, and the absorbance of the supernatant was determined at 532 nm. TBARS concentration was based on the molar extinction coefficient of malondialdehyde.

### Antioxidant capacity (DPPH assay)

Antioxidant substances of the serum were determined by 2,2-diphenyl-1-picrylhydrazyl (DPPH) radical assay [22,23]. Protein from serum samples (200 μL) was removed with acetonitrile (200 μL). Serum supernatant (without protein) was mixed with 970 μL of CH_3_OH and 5 μL of DPPH (10 mM in methanol), and rested at room temperature for 20 min, and centrifuged for 10 min at 10,000 rpm at 4°C. Absorbance of the supernatant was determined at 517 nm.

### Statistical analyses

Data were presented as means ± SD. Statistical analyses were done by Sigma Stat 3.1 software. Statistical comparisons of the groups were made by ANOVA One Way, followed by post hoc Tukey test for parameters with normal distribution, tested by Kolmogorov-Smirnov, or Student-Newman-Keuls for non-normal data. *P* value less than 0.05 was considered significant.

## Results

### Body weight and weight gain during the experimental period

There was no statistical difference in initial body weight, final body weight and weight gain between C and CH groups, and among the swimming groups, with or without hesperidin (CS, IS, CSH, ISH). But, the animals submitted to swimming (CS, IS, CSH, ISH) showed higher final body weight and weight gain in comparison to the animals without swimming (C and CH) (P < .05) (Table 1).

**Table 1:** Body weight of rats submitted to continuous or interval swimming with or without supplement

**Body weight**	**Group name **^**#**^					
	**C**	**CH**	**CS**	**CSH**	**IS**	**ISH**
*(n)*	*(10)*	*(10)*	*(10)*	*(10)*	*(10)*	*(10)*
Initial, g	408 ± 8.5	413 ± 4.1	404 ± 7.7	409 ± 16	413 ± 13	405 ± 4.1
Final, g	460 ± 19^a^	464 ± 9.8^a^	428 ± 7.6^b^	434 ± 19^b^	435 ± 7.8^b^	427 ± 11^b^
Weight Gain, g	52.0 ± 13.4^a^	51.4 ± 12.2^a^	24.0 ± 11.6^b^	25.3 ± 17.0^b^	21.8 ± 13.9^b^	22.0 ± 18.2^b^

^*#*^*C* negative control, *CH* positive control, *CS* continuous swimming, *CSH* continuous swimming + hesperidin, *IS* interval swimming, *ISH* interval swimming + hesperidin.

Results are expressed as mean ± SD.

^a, b^ Statistical differences among groups, indicated by different letters, were tested by Anova One Way, followed by Tukey test (*P* < 0.05).

### Glucose

There was a continuous decline of the serum glucose levels from the negative control group to the interval swimming group, as follow: negative control (C) > positive control (CH) > continuous swimming (CS) > continuous swimming + hesperidin (CSH) > interval swimming (IS) > interval swimming + hesperidin (ISH); suggesting a combined effect of hesperidin with swimming on the serum glucose. Statistically, glucose levels are higher for the C group, and lower for the ISH group, and all other groups with interval values (Table 2).

**Table 2:** Biochemical biomarkers of rats submitted to continuous or interval swimming with or without supplement

**Group name **^**#**^	**C**	**CH**	**CS**	**CSH**	**IS**	**ISH**
*(n)*	*(10)*	*(10)*	*(10)*	*(10)*	*(10)*	*(10)*
Glucose, mg/dL	93.9 ± 4.4^a^	91.2 ±2.5^ab^	88.2 ± 2.5^ab^	85.6 ± 3.9^bc^	81.5 ± 6.4^c^	75.3 ± 5.7^d^
Triglycerides, mg/dL	147 ± 15^a^	126 ± 13.1^b^	122 ± 17^b^	125 ±7.7^b^	115 ± 19^b^	108 ± 12^b^
Cholesterol, mg/dL	140 ± 22^ab^	118 ± 9.7^c^	120 ± 17^c^	106 ± 7.1^d^	146 ± 11.1^a^	125 ±10^b^
LDL-C, mg/dL	64.9 ± 15.6^a^	31.1 ± 14.4^b^	31.2 ± 17.9^b^	11.8 ±8.3^c^	55.2 ± 10.4^a^	32.6 ± 10.1^b^
HDL-C, mg/dL	45.4 ± 6.3^b^	61.2 ± 5.2^a^	63.9 ± 4.5^a^	72.0 ± 8.1^a^	68.2 ± 4.7^a^	70.6 ±4.9^a^
TBARS, μM	1.30 ± 0.45^a^	1.08 ± 0.31^a^	1.24 ± 0.29^a^	1.34 ± 0.18^a^	2.23 ± 1.37^b^	1.23 ± 0.33^a^
DPPH, % reduction	25.2 ± 4.5^b^	22.4 ± 3.3^b^	9.9 ± 3.9^a^	28.0 ± 3.6^c^	16.4 ± 1.5^b^	15.0 ± 13.4^b^

^*#*^*C* negative control, *CH* positive control, *CS* continuous swimming, *CSH* continuous swimming + hesperidin, *IS* interval swimming, *ISH* interval swimming + hesperidin.

Results are expressed as mean ± SD.

^a, b, c, d^ Statistical differences among groups, indicated by different letters, were tested by Anova One Way, followed by Tukey test for glucose, triglycerides, cholesterol, LDL-C, HDL-C, DPPH, and Student Newman-Keuls for TBARS (*P* < 0.05).

### Triglycerides

A 13% reduction of serum triglyceride levels was observed in the CH group compared to the C group. Among the exercised animals, with or without hesperidin (CS, CSH, IS, ISH), there were no observed differences on the triglyceride levels (Table 2).

### Total cholesterol and LDL-C

There was a decrease in serum total cholesterol levels of 15% in the CH group compared to the C group. The same response it was observed in the ISH group compared to its control IS (-15%) and in the CSH test related to its control CS (-11%) (Table 2). LDL-C levels were 52% lower in CH animals than in the C group. Similarly, LDL-C was 63% and 42% lower in the CSH and ISH groups, respectively than in their controls CS and IS (Table 2). These results follow the same trend found for total cholesterol, showing a markedly beneficial effect of hesperidin on the cholesterol metabolism.

### HDL-C

CH animals had high levels of blood serum HDL-C (35%) compared to the C group, while CS, IS, CSH and ISH also showed increased levels of HDL-C, suggesting that both hesperidin and exercise had a positive effect on HDL-C (Table 2).

### Lipid hydroperoxide (TBARS assay)

There was a marked increase of lipid peroxidation (around 60%) observed in IS rats in comparison to all groups. This result suggests that the intensity of the interval exercise promoted a higher oxidative stress, but this effect was attenuated by the hesperidin, as we observed in the ISH group (Table 2).

### Antioxidant capacity (DPPH assay)

Blood serum antioxidant capacity was over 2.8-fold higher in CSH compared to CS, but between the IS and ISH groups no difference was observed (Table 2).

## Discussion

Exercise training intervention is a low-risk conduct that has been designed as adjuvant treatment for chronic illnesses for many decades, but the combination of regular exercise with bioactive compounds to reduce chronic diseases risk factors has been a recent approach suggested in the literature [24,25]. This study tested whether continuous or interval swimming in addition to hesperidin supplementation in rats can improve biochemical and oxidative stress parameters related to chronic diseases.

The findings of the current investigation have shown that hesperidin supplementation in addition to continuous swimming (CSH) or interval swimming (HSE) improved biochemical and oxidative biomarkers in rats. Swimming training by itself, CS and IS groups, or in association with hesperidin, CSH and HSH groups, during four weeks improved glucose metabolism, decreased total cholesterol, LDL-C and triglycerides, and increased HDL-C. Furthermore, there was also an enhancement in the antioxidant capacity in the continuous swimming with hesperidin supplement, CSH group. Supplementation with hesperidin did not affect gain weight of rats during the 4-week period, but swimming training, continuous or interval, was an important factor in reducing the weight gain of all trained groups, suggesting that energy expenditure by exercise was the key factor to maintaining body weight [26].

Serum glucose concentration was significantly decreased when the animals were treated with hesperidin, whether associated with swimming or not, CSH, ISH and CH. Recent reviews have shown that regular exercise, continuous or interval, reduced serum glucose by improving insulin sensitivity [27,28], and high intense aerobic exercise induces an improvement of glucose control and adaptation in skeletal muscle [29]. According to the author, blood glucose was reduced by 13% over the 24-h period following training, and the postprandial glucose spikes were also reduced for several days afterwards. A recent study with rats that underwent interval swimming showed higher production of the glucose transporter GLUT-4, which is a determining factor for the transport and glucose uptake [30]. Moreover, hesperidin supplementation has important hypoglycemic effects by modulation of gene expression of hepatic enzymes such as glucokinase and glucose-6-fosfatase which are involved in the final step of catalyzing the gluconeogenesis and glycogenolysis, thus playing a role in regulating the homeostatic plasma glucose [31]. Others [32] have shown that isolated hesperidin in rats increased significantly the number of GLUT-2 and GLUT-4 carriers enhancing cellular signaling glucose and consequently reducing insulin resistance.

Increased levels of physical activity stimulate favorable changes on the levels of circulating lipoproteins, lowering the risks of metabolic disorders such as dyslipidemias, metabolic syndrome and diabetes [5-7]. These changes can vary according to the quantity and intensity of the training, which can decrease cholesterol and triglyceride levels and increase HDL-C [33,34], although a significant increase of HDL-C was more common with high-intensity resistance exercise [35]. On the other hand, citrus flavonoids such as hesperidin and naringin, chemically isolated or from citrus fruits and juices, have been associated with lower levels of LDL-C and triglycerides in humans [36] and animals [37,38]. Since the association between exercise training and hesperidin supplementation had not yet been addressed we investigated whether rats, submitted to swimming training alone (CS and IS) and in combination with hesperidin supplementation (CSH and ISH), would show increased beneficial effects on the lipid and lipoproteins metabolism.

In this study we observed that CH rats had a reduced level of serum triglycerides, suggesting that hesperidin is able to decrease the synthesis or catabolism of triglycerides-rich lipoproteins. A previous study [36] found that hesperidin supplement in subjects with hypertriglyceridemia (>150 mg/dL) dropped serum triglycerides, presumably because of the increase in triglyceride rich lipoproteins catabolism. On the other hand, it was shown [39] that hesperitin, the aglycon form of hesperidin, inhibited VLDL secretion *in vivo* and *in vitro* by inhibition of microsomal triglycerides transfer protein (MTP) activity, transcription of HMG CoA-reductase, ACAT activity and synthesis of Apo B, causing a 70% reduction in the secretion of hepatic ApoB-100/VLDL. Therefore, from these previous studies we can deduce that hesperidin was reducing both synthesis and catabolism of triglycerides. Except for the negative control group, the others (CH, CS, IS, CSH, ISH) showed lower levels of triglycerides, which suggested that hesperidin supplementation and swimming improved triglyceride metabolism, although the individual effects from exercise and supplement were not additive.

Regarding total cholesterol and LDL-C levels, we observed a marked reduction promoted by hesperidin in the CH, CSH and ISH groups in comparison to their controls (C, CS, IS) without supplementation. This result is corroborated by previous studies which showed that hesperidin lower plasma and liver cholesterol by inhibition of HMG CoA-reductase, ACAT and secretion of Apo B [39-41]. In addition hesperidin increased expression of the gene encoding the LDL receptor and its specific metabolism [42].

A recent study showed that either high-intensity or moderate-intensity exercise training reduced cardiovascular risk in rats with the metabolic syndrome. The authors found that both exercises improved endothelial function and blood pressure, increased HDL cholesterol, and reduced blood glucose. Also, the exercise reduced the impact of the metabolic syndrome and that the magnitude of the effect depends on exercise intensity [43]. Another study reported that acute resistance exercise in moderate or high intensity, as aerobic exercise, may have antiatherogenic effects, particularly throughout lipid profile modulation [44]. We observed in our study a concomitant increase of HDL-C on swimming groups (CS, IS) and on hesperidin-supplement groups (CH, CSH, ISH), but the effects were not additive.

HDL-C is a fraction of lipoprotein that carry cholesterol into the blood and is responsible for facilitating the reverse transport of cholesterol from peripheral tissues to the liver, where it is catabolized and excreted, and its function is recognized as antiatherogenic [45]. Although there are some controversies, it is well known that HDL-C levels is generally responsive to aerobic training and increases in a dose-dependent manner with increased energy expenditure [5]. Additionally the exercise intensity and duration are also associated with positive changes in the levels of HDL-C [43].

Because of the benefits that have been reported, regular physical exercise has been adopted as part of an overall strategy to normalize lipid profiles and to improve cardiovascular health [46]. However, it is questionable whether all physical exercise, despite the beneficial effects on lipid profile, might really be safe. It has been reported that exhaustive exercise, such as swimming, induces oxidative stress due to excessive oxygen reception and elevated production of ROS [47]. On the other hand, moderate regular exercise can have positive effects by upregulating the activities of antioxidant enzymes thereby reducing oxidative stress [48].

Regarding the oxidative stress and exercise, is well establish that prolonged or high-intensity exercises, such as interval training, increases the production of oxygen free radicals and lipid peroxidation which are related to oxidative damage to macromolecules in blood and skeletal muscle [49,50]. Therefore we evaluated the protective role of hesperidin, as an antioxidant compound, in continuous and interval exercise. No changes were observed in lipid peroxidation in the C, CH, CS, CSH groups, whereas there was a reduction of over 50% of lipid peroxidation triggered by the interval exercise (IS) with hesperidin supplementation in the ISH group. Previous study also attributed to hesperidin and naringin, and not to the vitamin C in orange juice, the effect of neutralizing the oxidative stress resulting from the ingestion of a pro-inflammatory high-fat, high-carbohydrate meal [51]. The continuous exercise increased the oxidative stress in animals that performed continuous swimming exercise (CS), however, the hesperidin supplement increased markedly (over 100%) the antioxidant capacity in the CSH group. Antioxidant capacity by hesperidin on other groups was unchanged (C, CH, CS, IS, ISH).

The antioxidant effects of the flavonoids quercetin [52] and eriocitrin [9] were also observed in swimming and running protocols, endorsing the idea that those flavonoids can prevent oxidative damage caused by exercise in the brain and liver, respectively. Another study attributed to isolated antioxidant compounds from legumes the capacity in inhibit xanthine oxidase (XO), the main enzyme related to the generation of free radicals during exercise [53], revealing beneficial health impacts as natural antioxidants of therapeutic interest, i.e. dietary [54]. Elsewhere the antioxidant activities of hesperidin and its metabolites have been shown by the increase of antioxidant enzymes and the decrease of oxidative stress [14,55].

## Conclusions

In summary, the findings of the present study have shown that hesperidin supplementation per se or in combination with swimming exercise protocols, continuous and interval, potentiates improvement of the biochemical profile and antioxidant biomarkers evidencing that the use of citrus flavonoids may be beneficial to reduce risk factors for metabolic and cardiovascular diseases. Moreover, hesperidin supplementation, in conjunction with continuous swimming, presented hypolipidemic effects and could be useful as an antioxidative compound to protect against oxidative damages during this type of exercise; on the other hand, hesperidin plus interval swimming exercise can help reduce increased levels of glucose in the blood serum.

## Abbreviations

ACAT: Acetyl-Coenzyme A acetyltransferase; Apo B: Apolipoprotein B; C: Negative control group, no swimming and no supplement; CH: Positive control group, no swimming plus hesperidin supplement; CH3OH: Methanol; CS: Continuous swimming group, continuous swimming and no supplement; CSH: Continuous swimming plus hesperidin group, continuous swimming plus hesperidin supplement; DPPH: 2,2-diphenyl-1-picrylhydrazyl; GLUT-2: Glucose transporter type 2; GLUT-4: Glucose transporter type 4; HDL-: High density lipoprotein-cholesterol; HMG CoA-reductase: 3-hydroxy-3-methyl-glutaryl-CoA reductase; IS: Interval swimming group, interval swimming and no supplement; ISH: Interval swimming plus hesperidin group, interval swimming plus hesperidin supplement; LDL-C: Low density lipoprotein-cholesterol; MDA: Malondialdehyde; MTP: Microsomal triglycerides transfer protein; SDS: Sodium dodecyl sulphate; TBA: Thiobarbituric acid; TBARS: Thiobarbituric acid-reactive substances.

## Competing interests

The results of the present study do not constitute endorsement of any products by the authors or by ACMS or other organizations. The authors declare that we do not have any conflicts of interest and that the source of funding is independent of the objectives and results found in this study.

## Authors’ contributions

The authors David de Oliveira and Grace Dourado participated in the collection of data, biochemical evaluation and statistical analysis. The interpretation of data and writing of the text were accomplished by all authors, including Thais Cesar, who was the mentor of this work. All authors have seen and approved the final version of this paper.
